# Accelerometer-based assessment of physical activity within the Fun For Wellness online behavioral intervention: protocol for a feasibility study

**DOI:** 10.1186/s40814-019-0455-0

**Published:** 2019-05-31

**Authors:** Nicholas D. Myers, Seungmin Lee, André G. Bateman, Isaac Prilleltensky, Kimberly A. Clevenger, Karin A. Pfeiffer, Samantha Dietz, Ora Prilleltensky, Adam McMahon, Ahnalee M. Brincks

**Affiliations:** 1 Department of Kinesiology, Michigan State University, 201 IM Sports Circle Building, 308 W. Circle Drive, East Lansing, MI 48824 USA; 2 School of Education and Human Development, University of Miami, Coral Gables, USA; 3 Department of Epidemiology and Biostatistics, Michigan State University, East Lansing, USA

**Keywords:** Self-efficacy theory, Well-being, Validity, Acceptability, E-health, M-health

## Abstract

**Background:**

Fun For Wellness (FFW) is an online behavioral intervention designed to promote growth in well-being and physical activity by providing capability-enhancing learning opportunities to participants. The conceptual framework for the FFW intervention is guided by self-efficacy theory. Evidence has been provided for the efficacy of FFW to promote self-reported free-living physical well-being actions in adults who comply with the intervention. The objective of this manuscript is to describe the protocol for a feasibility study designed to address uncertainties regarding the inclusion of accelerometer-based assessment of free-living physical activity within the FFW online intervention among adults with obesity in the United States of America (USA).

**Method:**

The study design is a prospective, double-blind, parallel group randomized pilot trial. Thirty participants will be randomly assigned to the FFW or usual care (UC) group to achieve a 1:1 group (i.e., FFW:UC) assignment. Recruitment of participants is scheduled to begin on 29 April 2019 at a local bariatric services center within a major healthcare organization in the Midwest of the USA. There are five eligibility criteria for participation in this study: (1) between 18 and 64 years old, (2) a body mass index ≥ 25.00 kg/m^2^, (3) ability to access the online intervention, (4) the absence of simultaneous enrollment in another intervention program promoting physical activity, and (5) willingness to comply with instructions for physical activity monitoring. Eligibility verification and data collection will be conducted online. Three waves of data will be collected over a 13-week period. Instruments designed to measure demographic information, anthropometric characteristics, acceptability and feasibility of accelerometer-based assessment of physical activity, self-efficacy, and well-being will be included in the study. Data will be analyzed using descriptive statistics (e.g., recruitment rates), Pearson’s correlation coefficient, Bland-Altman analyses, and inferential statistical models under both an intent to treat approach and a complier average causal effect approach.

**Discussion:**

Results are intended to inform the preparation of a future definitive randomized controlled trial.

**Trial registration:**

ClinicalTrials.gov, NCT03906942, registered 8 April 2019.

**Trial funding:**

The Erwin and Barbara Mautner Charitable Foundation and the Michigan State University College of Education.

**Electronic supplementary material:**

The online version of this article (10.1186/s40814-019-0455-0) contains supplementary material, which is available to authorized users.

The World Health Organization (WHO) estimates that there are 650 million adults with obesity and that the number of adults with obesity has tripled since 1975 [1]. Obesity is a risk factor for major non-communicable diseases such as cardiovascular disease, type II diabetes, musculoskeletal disorders, and some cancers [2]. To reduce the prevalence of adults with obesity, the WHO recommends that individuals limit energy intake from low-quality food sources (e.g., highly processed foods high in fat), increase energy intake from high-quality food sources (e.g., raw vegetables), and engage in a recommended amount of physical activity for health [1]. Examples of a recommended amount of physical activity (counting only those physical activities that you do for at least 10 min at a time) for adults include at least 150 min per week of moderate physical activity, at least 75 min per week of vigorous physical activity, or an equivalent combination of the two recommendations listed above [3, 4]. There is evidence, however, that very few (e.g., < 5%) adults with obesity may engage in a recommended amount of physical activity [5]. Fortunately, there also is evidence that well-designed cognitive-behavioral interventions can successfully promote physical activity in obese adults [6].

## Self-efficacy theory

Self-efficacy theory resides within the more general social cognitive theory [7]. In social cognitive theory, individuals are regarded as proactive agents in the regulation of their cognition, motivation, actions, and emotions. Self-efficacy judgments occupy a central role in self-efficacy theory and are defined as domain-specific beliefs held by individuals about their ability to successfully execute differing levels of performance given certain situational demands [8]. The formation of self-efficacy beliefs is believed to rely upon the cognitive processing of diverse sources of efficacy information that can be categorized as follows: past performance accomplishments, vicarious experiences, verbal persuasion, and physiological and/or emotional states. Two proposed outcomes of self-efficacy beliefs are an individual’s thought patterns (e.g., goal setting, worry, and attributions) and behavior (e.g., challenges undertaken, effort expended on challenges undertaken, and persistence in the face of difficulties that arise during challenges undertaken). A necessary condition for valid testing of self-efficacy theory is concordance between the domain-specific self-efficacy beliefs and the proposed outcomes of the self-efficacy beliefs of interest [8]. There is a rich literature on the potential importance of increasing self-efficacy for physical activity as a mechanism for promoting physical activity in adults [9, 10].

### Physical activity

Physical activity has been defined as bodily movement produced by skeletal muscles that require energy expenditure [11]. There is evidence that insufficient physical activity increases the risk of several major non-communicable diseases (e.g., cardiovascular disease, type II diabetes, and some cancers) in adults worldwide [12]. Unfortunately, insufficient physical activity in the adult population is a global pandemic [13, 14]. Successfully addressing this pandemic will require ongoing and wide implementation of a variety of intervention strategies (e.g., community-wide, informational, behavioral, social, policy, and built environment) at multiple levels of society (e.g., individual, neighborhood, municipality, country) across the globe [15, 16]. At the individual-level, there is evidence that behavioral interventions designed to promote physical activity by focusing on personal psychological attributes (e.g., self-efficacy beliefs) can be effective [9, 10]. Delivering a physical activity intervention online has been shown to be an effective mode of delivery [17, 18] that also may allow for efficient scaling up of an intervention [16]. Thus, a readily scalable online behavioral intervention that effectively promotes physical activity in adults with obesity by providing self-efficacy enhancing opportunities may be useful in regard to responding to a global pandemic (i.e., physical inactivity) in an at-risk population (i.e., adults with obesity).

### Activity monitors

Wider use of activity monitors (e.g., pedometers and/or accelerometers) to assess physical activity in field-based research on adults began to be steadily advocated for more than a decade ago [19–22]. A primary reason that the use of activity monitors was advocated for was to address long-standing concerns regarding potential limitations for the validity of physical activity estimates based on self-report [23–26]. Accelerometers generally were recommended in field-based research when the indicator of physical activity was to include a dimension of exercise duration and/or intensity (e.g., time spent in moderate to vigorous physical activity (MVPA)) and the cost of the activity monitor was not prohibitive [19–22]. Recent reports suggest that the use of accelerometers to assess physical activity in field-based research on adults may be increasing [27–29]. The scientific importance of this trend is reinforced by troubling findings that suggest only small to moderate relative agreement and large absolute disagreement between estimates of physical activity based on self-report versus accelerometry [30]. Moreover, there is evidence that even some commercial-grade accelerometers that are relatively modest in cost can provide valid estimates of free-living (i.e., occurring outside of controlled laboratory conditions) physical activity among adults [31].

The use of activity monitors in physical activity behavioral interventions for adults with obesity has been recommended based on two key findings from a recent meta-analysis of 11 randomized controlled trials (RCTs) [32]. The first key finding was that physical activity behavioral interventions with an activity monitor were shown to increase physical activity in adults with obesity. The second key finding was that it appears that adding an activity monitor to an existing physical activity behavioral intervention (previously without an activity monitor) may increase the magnitude of the effect of the intervention on physical activity in adults with obesity. These two key findings, along with a synthesis of 14 relevant RCTs (11 of which were included in the aforementioned meta-analysis), led the Community Preventive Services Task Force to recommend that physical activity interventions for adults with obesity should include activity monitors and behavioral instructions (e.g., web-based education) [33]. Furthermore, the Community Preventive Services Task Force recommends that physical activity interventions for adults with obesity should promote physical activity within a more broadly focused weight management program where there is access to a health care provider [33].

### Feasibility

Assessing the feasibility of participants wearing an accelerometer as requested by the research team for a particular field-based study is recommended [21]. Two large studies conducted in the United States of America (USA) that assessed the feasibility of adult participants wearing an accelerometer as instructed are the 2003–2004 National Health and Nutritional Examination Survey (NHANES) [22] and the 2009–2013 physical activity ancillary study within the Reasons for Geographic and Racial Differences in Stroke (REGARDS) study [34, 35]. While both of these studies provide models for assessing the feasibility of adult participants wearing an accelerometer as requested by the research team, neither of these studies was an online behavioral intervention. The feasibility of participants wearing an accelerometer as requested in a longitudinal online behavioral intervention may pose unique challenges stemming from an inability to hand deliver an accelerometer (as in the 2003–2004 NHANES study) and the lack of an established research-based relationship with participants (as in the 2009–2013 physical activity ancillary REGARDS study).

### Fun For Wellness (FFW)

FFW is an online behavioral intervention designed to promote growth in well-being and physical activity by providing capability-enhancing learning opportunities to participants [36, 37]. The conceptual framework for the FFW intervention is guided by self-efficacy theory [8]. The target audience of the FFW intervention is the adult population who would be comfortable with the online platform within which the intervention is delivered. There is an emerging literature on the efficacy and the effectiveness of the FFW intervention [36–39].

### 2015 FFW efficacy trial

**Fig. 1:**
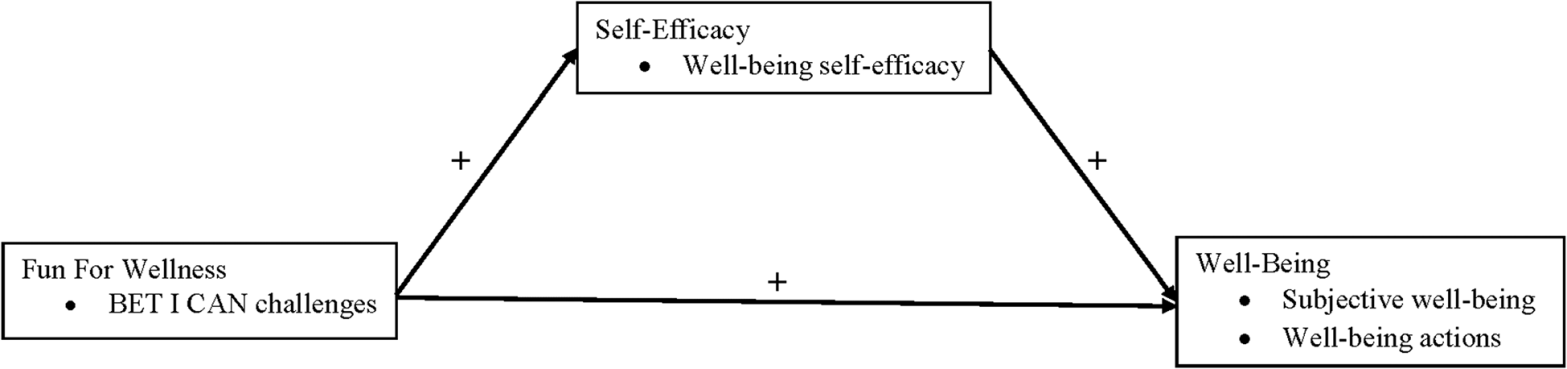
The conceptual model that guided the 2015 Fun For Wellness efficacy trial [36]

A RCT completed in 2015, hereto forward referred to as the 2015 FFW efficacy trial, provided the initial test of the FFW intervention to promote well-being [36]. Figure 1 depicts the conceptual model that guided the 2015 FFW efficacy trial. The FFW intervention (i.e., engagement with the BET I CAN challenges) was conceptualized as exerting both a positive direct effect and a positive indirect effect through self-efficacy (i.e., well-being self-efficacy), on well-being (i.e., subjective well-being and well-being actions). Each of the constructs depicted in Fig. 1 will be defined in the “Methods” section of this manuscript.

Data collection for the 2015 FFW efficacy trial occurred within a relatively controlled environment (i.e., adult employees at a major research university in the USA). Results from the 2015 FFW efficacy trial provided some initial evidence for the efficacy of FFW to promote well-being self-efficacy [38]; interpersonal, community, psychological and economic subjective well-being [36]; and interpersonal and physical well-being actions [39]. The protocol for the feasibility study to be described in this manuscript seeks to follow up on the initial evidence provided in the 2015 FFW efficacy trial for the FFW intervention to promote self-reported free-living physical well-being actions (e.g., engagement in physical activity) in adults who comply with the intervention [39].

### 2018 FFW effectiveness trial

**Fig. 2:**
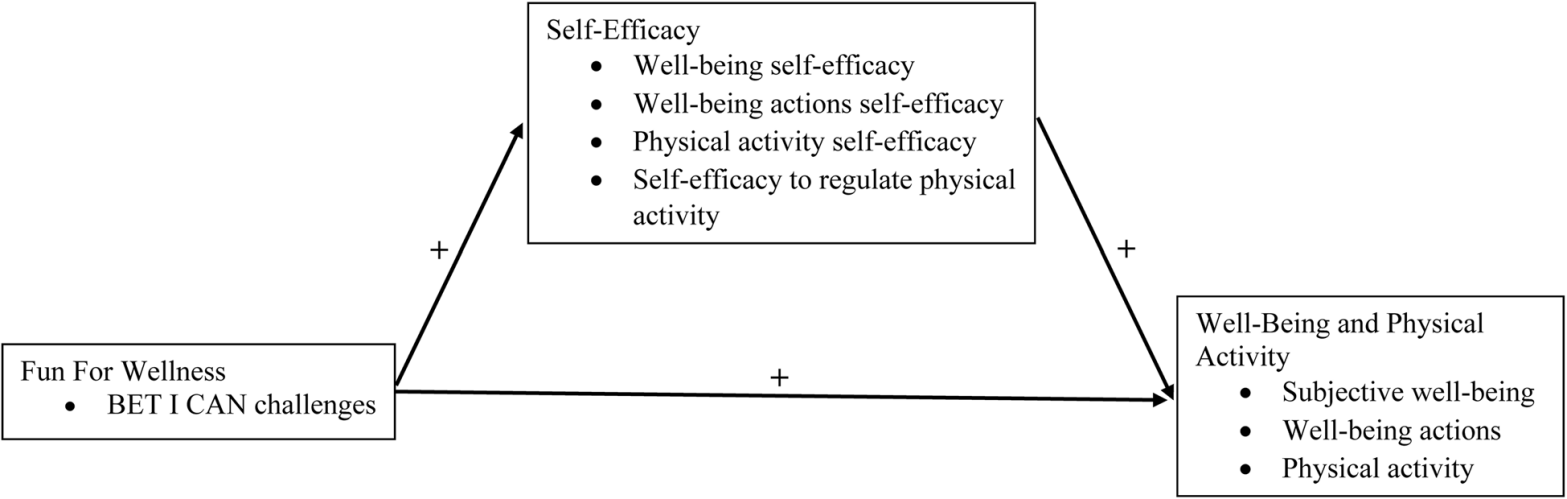
The conceptual model that guided the 2018 Fun For Wellness effectiveness trial [37]

A RCT designed to provide an initial evaluation of the effectiveness of the FFW intervention to increase well-being and free-living physical activity in an adult population with obesity enrolled approximately 900 participants and hereto forward is referred to as the 2018 FFW effectiveness trial [37]. Figure 2 depicts the conceptual model that guided the 2018 FFW effectiveness trial. The FFW intervention was conceptualized as exerting both a positive direct effect and a positive indirect effect through self-efficacy, on well-being and physical activity. Four constructs—well-being actions self-efficacy, physical activity self-efficacy, self-efficacy to regulate physical activity, and physical activity—were added to the conceptual model in the 2018 FFW effectiveness trial (i.e., compare Fig. 2 to Fig. 1) based on the findings from the 2015 FFW efficacy trial. Each of the additional constructs in Fig. 2 will be defined in the “Methods” section of this manuscript.

The 2018 FFW effectiveness trial was built upon the 2015 FFW efficacy trial by having a stronger focus on promoting physical activity and by recruiting participants from a relatively uncontrolled context (i.e., via an online panel recruitment company) [37]. The increased focus on physical activity in the 2018 FFW effectiveness trial was manifest by a particular emphasis on engaging in a recommended amount of physical activity for health (i.e., time spent in MVPA) in the introduction to the BET I CAN challenges. The 2018 FFW effectiveness trial also was built upon the 2015 FFW efficacy trial by more thoroughly assessing self-reported physical activity with the long form (i.e., 27-items) of the international physical activity questionnaire (IPAQ) [40, 41]. The aforementioned long-standing concerns regarding potential limitations for the validity of physical activity estimates based on self-report, however, are relevant to both the 2015 FFW efficacy trial and the 2018 FFW effectiveness trial. That is, neither the 2015 FFW efficacy trial nor the 2018 FFW effectiveness trial used objective instrumentation (e.g., accelerometry) to measure physical activity.

### Proposed study

The feasibility study proposed in this paper will be the first study to use accelerometry within the FFW online behavioral intervention. The objective of this manuscript is to describe the protocol for a feasibility study designed to address uncertainties regarding the inclusion of accelerometer-based assessment of free-living physical activity within the FFW online intervention among adults with obesity in the USA. Four specific aims will be investigated.

#### Aim 1

To determine if accelerometer-based assessment of physical activity *can* be used within the FFW intervention.

#### Aim 2

To determine if accelerometer-based assessment of physical activity *should* be used within the FFW intervention.

#### Aim 3

To determine *how* to implement accelerometer-based assessment of physical activity within the FFW intervention. Aim 3 assumes a positive response to Aim 2.

#### Aim 4

To provide a preliminary effect size estimate for each direct effect depicted in the conceptual model (see Fig. 2) for the FFW online intervention (e.g., FFW → Physical Activity).

Pursuit of these four specific aims is based on a general conceptual framework for feasibility and pilot studies in preparation for a future definitive RCT [42–44]. Within this conceptual framework, a randomized pilot trial is a type of a feasibility study and a feasibility study may include a focus on the acceptability of an intervention [42–44].

## Methods/design

### Ethical approval

All procedures in this study involving human participants will be in accordance with the ethical standards of the institutional and/or national research committee and with the 1964 Helsinki declaration and its later amendments or comparable ethical standards. The institutional review board at Michigan State University provided necessary permission to conduct this study on 1 April 2019, STUDY00002012. Table 1 provides the SPIRIT flow diagram, which includes a list of the assessments to be taken during the course of this study. A populated SPIRIT checklist is provided in the Additional file 1.

**Table 1:** The SPIRIT flow diagram for the Fun For Wellness accelerometer feasibility study

Time point	Study period							
	Enrolment	Allocation	Post-allocation					Close-out
	*−t*_*1*_	0	w1 Weeks 1–3	w2 Weeks 3–7	w3 Weeks 7–13			*t*_*x*_
Enrolment								
Eligibility screen	X							
Informed consent	X							
Allocation		X						
Interventions								
*Fun For Wellness*				X				
*Usual care*			X	X	X			
Assessments								
Gender			X					
Age			X					
Race			X					
Education-level			X					
Marital status			X					
Annual income			X					
Zip code			X					
Height			X	X	X			
Weight			X	X	X			
Physical activity			X	X	X			
Acceptability of accelerometer-based assessment of physical activity			X	X	X			
Self-efficacy to comply			X					
Well-being self-efficacy			X	X	X			
Well-being actions self-efficacy			X	X	X			
Physical activity self-efficacy			X	X	X			
Self-efficacy to regulate physical activity			X	X	X			
Subjective well-being			X	X	X			
Well-being actions			X	X	X			

### Study design

**Fig. 3:**
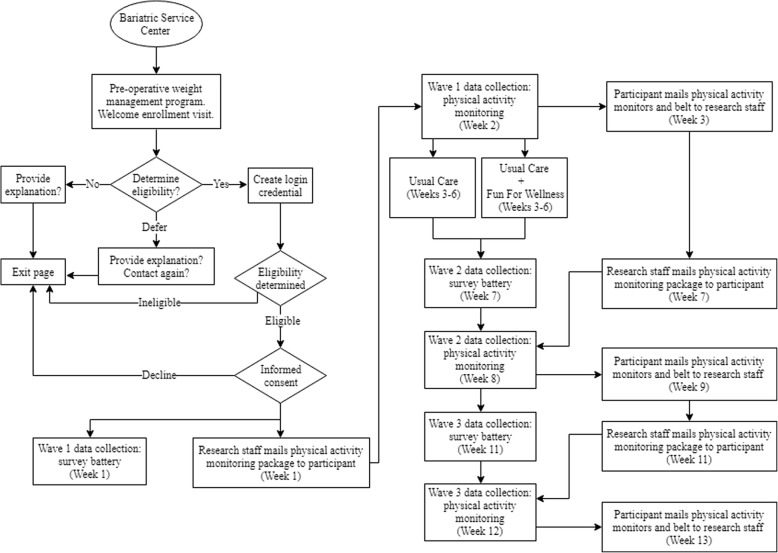
Flow chart for recruitment of participants throughout data collection

The study design is a prospective, double-blind, parallel group randomized pilot trial. Recruitment of participants is scheduled to begin on 29 April 2019 at a local bariatric services center, hereto forward referred to as the center, within a major healthcare organization in the Midwest of the USA. Eligibility verification and data collection will be conducted online. Three waves of data collection (i.e., at W1, W2, and W3) will be collected over a 13-week period. Figure 3 provides a flow chart for recruitment of participants throughout data collection. Instruments designed to measure demographic information, anthropometric characteristics, acceptability and feasibility of accelerometer-based assessment of physical activity, self-efficacy, and well-being will be included in the study.

### Recruitment

Participants will be recruited from a relatively controlled local context to allow for the possibility that university-based researchers (i.e., research staff) may need to follow up with participants during the course of the study to address unforeseen areas of uncertainty that often arise during a feasibility study [41–43]. Patients who are candidates for bariatric surgery and are enrolling in the preoperative weight management program provided by the center will be recruited for possible participation in this study. Successful enrollment in the preoperative weight management program provided by the center requires a medical history intake and a physical exam (e.g., medical clearance to engage in physical activity).

Recruitment for possible participation in this study will be extended to each patient near the end of the welcome enrollment visit at the center. At this time, a center staff member will provide the patient with login information to the secure recruitment page on the FFW website which can then be accessed by either a laptop located in the center (and provided by the research staff) or via their own device (e.g., smartphone). The patient will then be asked to independently engage with the FFW website in the waiting room. The center staff member will, however, inform the patient that both they and the research staff are available to respond to any questions that may arise during recruitment and throughout the online pilot study if necessary. Additionally, a member of the research staff may be available in the waiting room (depending on related scheduling issues) to assist the patient with website and/or study-related questions. Research staff will have regular contact with center staff throughout the study to discuss any questions or concerns that may arise.

The recruitment page on the FFW website will provide a brief description of the study and will ask the patient if they are interested in determining if they are eligible for participation in the study. Response options will include “yes”, “no”, and “not now, maybe later” (i.e., “defer”) based on previous research [34]. Patients who select “no” will be invited to provide a brief explanation for their response. Patients who “defer” will be invited to provide a brief explanation for their response and then will be asked if they would like to be contacted again in the future to discuss possible participation in this study. Patients who select “no” or “defer” will be taken to the exit page. The exit page will thank an individual for their time and ask them to check out with center staff. Patients who select “yes” will be asked to create a unique and secure login credential by providing a phone number and an e-mail address and creating a password. Patients who select “yes” will also be asked if research staff can send study-related text messages to the phone number they provided. After the login credential is verified by the patient, the potential participant will be screened to determine their eligibility for participation in the study.

### Eligibility

There are five eligibility criteria for participation in this study. Values for each of the eligibility criteria will be based on self-report by the potential participant. The first two eligibility criteria focus on physical characteristics of the potential participant. The first eligibility criterion is being between 18 and 64 years old. A justification for this criterion is that our target age-based population is adults (i.e., 18–64 years) and not older adults (i.e., 65 years and above) based on evidence-based age groupings for global recommendations on physical activity for health [3, 4]. The second eligibility criterion is a body mass index (BMI) ≥ 25.00 kg/m^2^. The BMI criterion in this study includes overweight (i.e., 25.00–29.99 kg/m^2^) and obese (i.e., ≥ 30.00 kg/m^2^) categories, consistent with many physical activity interventions in adults with obesity [6, 32, 33]. A justification for this criterion is the need to promote physical activity in a BMI-based population in which few individuals may meet public health guidelines for physical activity [5].

The third and the fourth eligibility criteria focus on the interaction of the potential participant and the online intervention. The third eligibility criterion is the ability to access the online intervention. This criterion will be assessed by asking each individual to confirm that they will have access to a technological device (e.g., computer, smartphone) that can access the online intervention via a web browser throughout the study. The fourth eligibility criterion is the absence of simultaneous enrollment in another intervention program promoting either well-being or physical activity. This criterion will be assessed by requiring each individual to confirm that they will not be enrolled in another formal intervention program (not counting the pre-operative weight management program provided by the center) promoting either well-being or physical activity during the FFW study period. A justification for this criterion is a reduction in the likelihood of confounding the results from the current study with results that may be due to enrollment in other programs.

The final eligibility criterion is a willingness to comply with instructions for physical activity monitoring. This criterion will be assessed by asking each potential participant if they are willing to wear an adjustable nylon belt around their waist with two accelerometers attached to it for three 7-day intervals and complete a daily log sheet regarding wear time during each of the three 7-day intervals over a 13-week period. A justification for this criterion is based on the physical activity ancillary REGARDS study where a similar question was asked to potential participants at screening [34]. Individuals who indicate a willingness to comply with instructions for physical activity monitoring will be asked to provide (a) an estimate of their waist circumference for belt sizing and (b) a mailing address where the physical activity monitors can be sent. Individuals who do not meet one or more of the eligibility criteria will be informed that they are ineligible for the study at this time and then will be taken to the exit page.

### Informed consent

Potential participants who meet all of the eligibility criteria will immediately be presented with the informed consent form to read and sign electronically. Individuals who click “decline to consent” will be locked out of the intervention and will be taken to the exit page. Individuals who do not respond will be taken to the exit page and subsequently will be contacted by research staff and asked if they would like to either “decline to consent” or “consent”. Individuals who click “consent” will be informed that they are enrolled as a participant in this study.

### Wave (W) 1 survey battery

Immediately after enrolling in this study, participants will be taken to a new page to complete the W1 survey battery. After completing the W1 survey battery, participants will be informed that a physical activity monitoring package (PAMP) will be sent to their mailing address within 1 week and then will be taken to the exit page. The specific content of both the W1 survey battery (e.g., demographics) and the PAMP will be detailed in the “Data collection” section of this manuscript.

### Sample size

Thirty participants will be targeted for enrollment in this study. There are three rationales for the target sample size. First, the target sample size fits well within the range of sample sizes often observed in pilot and feasibility trials [45]. Second, and with regard to aim 1 through aim 3 of this study, the target sample size is consistent with sample sizes observed in similar research [31, 46–48]. Third, and with regard to Aim 4 of this study, the target sample size is based on budgetary constraints and not a sample size determination for a desired level of statistical power [42–44]. Budgetary constraints preclude enrollment of more than 30 participants in this study.

### Randomization

Following completion of the W1 survey battery, participants will be randomly assigned to either the FFW group or the usual care (i.e., UC) group by the code programmed within the FFW website that is specified to achieve a 1:1 group (i.e., *n*_FFW_ = 15_,_ *n*_UC_ = 15) assignment.

Participants, center staff, and research staff will be blinded to participant group assignment.

#### Usual care

Participants assigned to the UC group (i.e., UC participants) will proceed through the preoperative weight management program provided by the center. The login credential for each UC participant will, however, provide access to a secure website to complete data collection (i.e., a survey battery and physical activity monitoring) at W1, W2, and W3. UC participants will have the opportunity to receive up to $30 worth of Amazon electronic gift cards, which is similar to previous research [36, 37]. Specifically, UC participants will receive $10 for completing the W1 survey battery and W1 physical activity monitoring, $10 for completing the W2 survey battery and W2 physical activity monitoring, and $10 for completing the W3 survey battery and W3 physical activity monitoring. UC participants will be given 4 weeks of 24 h access to the FFW online intervention approximately 1 month after data collection for this study is closed.

#### Fun For Wellness

Participants assigned to the FFW group (i.e., FFW participants) will proceed through the preoperative program provided by the center and will be given 4 weeks of 24 h access to the FFW online intervention during data collection for this study. The login credential for each FFW participant will provide access to both the FFW intervention and to a secure website to complete data collection at W1, W2, and W3. FFW participants will have the opportunity to receive a total of up to $45 worth of Amazon electronic gift cards, which is similar to previous research [36, 37]. Specifically, FFW participants will receive $10 for completing the W1 survey battery and W1 physical activity monitoring; $25 for completing the W2 survey battery, W2 physical activity monitoring, and at least 30 post-introductory BET I CAN challenges; and $10 for completing the W3 survey battery and W3 physical activity monitoring. The remuneration plan at W2 is linked to completing post-introductory BET I CAN challenges to encourage compliance with the FFW intervention as suggested in previous research [36, 37].

#### BET I CAN challenges

Self-efficacy theory [8] provided the theoretical framework that guided the creation of capability-enhancing learning opportunities (i.e., BET I CAN challenges) for FFW participants to engage with. The capability-enhancing learning opportunities provided to participants come in the form of 152 interactive and scenario-based challenges organized in the online environment by the BET I CAN acronym [36]. The *b*ehavior-focused challenges are intended to increase a participant’s capabilities to set a goal and to create positive habits [49]. The *e*motion-focused challenges are intended to increase a participant’s capabilities to cope with negative emotions and to cultivate positive emotions [50]. The *t*hought-focused challenges are intended to increase a participant’s capabilities to challenge negative assumptions and to create a new narrative for their life [51]. The *i*nteraction-focused challenges are intended to increase a participant’s capabilities to communicate and connect with others [52]. The *c*ontext-focused challenges are intended to increase a participant’s capabilities to read cues and to change cues in the environment [53]. The *a*wareness-focused challenges are intended to increase a participant’s capabilities to know herself/himself and to know the issue [54]. The *n*ext step-focused challenges are intended to increase a participant’s capabilities to make a plan and to stick with a plan [55]. In summary, the BET I CAN challenges in the FFW intervention provide opportunities for a participant to increase his or her capabilities to organize and execute actions that may increase their well-being and physical activity [36, 37].

### Data collection

#### Study timeline

Three waves of data will be collected over a 13-week period (see Fig. 3), which is similar to both the 2015 FFW efficacy trial [36] and the 2018 FFW effectiveness trial [37]. Data collection at W1 will include the W1 survey battery and W1 physical activity monitoring. The W1 survey battery will be completed in week 1. The W1 physical activity monitoring will be completed in week 2. Participants will return physical activity monitoring items (to be detailed in the physical activity monitoring section) to the research staff in week 3. The intervention will be delivered in week 3 through week 6. Data collection at W2 will include the W2 survey battery and W2 physical activity monitoring. The W2 survey battery will be completed in week 7. The W2 physical activity monitoring will be completed in week 8. Participants will return physical activity monitoring items to the research staff in week 9. Data collection at W3 will include the W3 survey battery and W3 physical activity monitoring. The W3 survey battery will be completed in week 11. The W3 physical activity monitoring will be completed in week 12. Participants will return physical activity monitoring items to the research staff in week 13. The timeline for this study is similar to timelines used in other physical activity interventions in adults with obesity [6, 32, 33].

#### W1, W2, and W3 physical activity monitoring

Instruments designed to measure physical activity and the acceptability and feasibility of accelerometer-based assessment of physical activity will be included in the PAMP. Research staff will prepare and send the PAMP to each participant via regular USA mail approximately 1 week before physical activity monitoring is scheduled to occur. A commercial-grade (Fitbit Zip) and a research-grade (Actigraph wGT3X-BT) accelerometer will be initialized and attached to an adjustable nylon belt to measure physical activity objectively. The order of the two monitors (i.e., which monitor is medial or lateral) will be randomized to account for small differences by wear location. The same two monitors will be provided to each participant at each time point to limit inter-monitor variability. Throughout the study duration, no app, firmware, or software updates will be allowed and one laptop computer will be used for all monitors, to ensure consistent clock times for data analysis. The primary measure of physical activity in this study will be the average minutes per day of MVPA based on both widely accepted recommendations for health-enhancing physical activity [3, 4] and the particular physical activity emphasis manifest in the FFW intervention [37].

Consistent with previous research [34], a brief cover letter, written and pictorial wear instructions, a daily log sheet regarding wear time, protocol checklist, and one preaddressed postage-paid, padded, return envelope (regular USA mail) will be included in the PAMP. In the cover letter, participants will also be directed to use their login credential to access electronic copies of the paper-based documents in the PAMP. Wear instructions will include the following: ensure that the belt is snug around the waist and that monitors are placed over the right hipbone crest at the anterior axillary line during wear time; begin to wear the monitors upon awakening the day after receipt for a seven-day interval; remove the monitors when sleeping or during water intensive activities (e.g., swimming, bathing) and reattach the monitors upon awakening or at cessation of water intensive activities; complete the daily log sheet online with date and time the monitors are put on and taken off each day; and return the monitors and belt on the day after a 7-day interval via the preaddressed postage-paid, padded, return envelope provided. On the day after a 7-day interval, participants will receive a reminder by e-mail and/or phone to return the belt and the monitors. At this time, participants will also be prompted to complete instruments designed to measure the acceptability of accelerometer-based assessment of physical activity and self-reported physical activity.

Upon return of the belt and the monitors the research team will follow a protocol based on previous research [34]. Data will be downloaded using a standard universal serial bus port. Belts will be laundered. Batteries in the monitors will be charged or changed if necessary. Monitors will be reinitialized for use. Within a few weeks of the return of the monitors, participants who provide usable data (to be described in the section on the ActiGraph wGT3X-BT) will be e-mailed (or mailed via regular mail if an e-mail address is unavailable) a preliminary estimate of their wear time and average minutes per day of MVPA in relation to broad categories of recommended MVPA per week (i.e., 0–4.21 min/day = minimal; 4.21–21.36 min/day = less than recommended; ≥ 21.36 min/day = meeting recommendation of 150 min/week). Participants who do not provide usable data will be informed that their average minutes per day of MVPA cannot be estimated due to insufficient data.

#### Fitbit Zip

The Fitbit Zip (San Francisco, CA, USA) is a tri-axial commercial-grade accelerometer that will be used to measure physical activity objectively. There is evidence for the validity of physical activity measures produced by the Fitbit Zip in both treadmill-based [31, 56] and free-living [31] activities in adults. Average minutes per day of MVPA will be determined using the Fitbit application, which uses a proprietary algorithm to estimate *fairly active minutes* and *very active minutes*, consistent with previous research [47, 48].

The cost of a Fitbit Zip is approximately 60 USA dollars. The Fitbit Zip can store data up to 7 days and sync wirelessly with the Fitbit mobile application up to a 20-ft range. A Fitbit website account will be established for each Fitbit Zip, but a participant will not be able to access the account [47]. The default settings of the Fitbit Zip (e.g., goals) will be removed because these settings may be inappropriate for adults with obesity [57]. Removing these settings also reduces the likelihood of confounding effects from behavior change techniques built into activity monitors [58]. The only physical activity display that a participant will be able to view while wearing this device will be the number of steps taken because this information cannot be programmed to be hidden.

#### ActiGraph wGT3X-BT

The ActiGraph wGT3X-BT (Pensacola, FL, USA) is a tri-axial research-grade accelerometer that will be used to measure physical activity objectively. ActiGraph devices have been used extensively as a reference (e.g., gold-standard) device to measure free-living physical activity objectively in adults [29]. Monitors will be initialized to collect raw acceleration data at 30 Hz using ActiLife software (version 6.13.3). Upon download, data will be re-integrated to 60-s epochs, and non-wear time will be defined as ≥ 90 continuous minutes of zero counts, with allowance for 2 min of acceleration that are preceded and followed by at least 30 min of continuous zeros [59]. Usable data will be defined as follows: (a) a log sheet with a valid start date is submitted, (b) a monitor is worn for at least 4 days (including one weekend day) with at least 10 h of valid wear time per day, and (c) no evidence of monitor error (e.g., activity counts > 20,000 or lengthy strings of repeated activity counts). Unusable data will be treated as missing data. Average minutes per day of MVPA will be calculated based on established cut points (e.g., > 1952 counts per minute) [60].

The cost of an ActiGraph wGT3X-BT is approximately 225 USA dollars. The ActiGraph wGT3X-BT can store data up to 43 days and sync wirelessly with the ActiLife mobile application; however, the wireless function will be disabled to maximize battery life. A participant will not be able to view any physical activity displays while wearing this device. The pairing of the ActiGraph wGT3X-BT with the Fitbit Zip is based on previous research where at least a moderately high correlation and a moderate level of agreement between measures of average minutes per day of MVPA produced by these two accelerometers has been observed [47, 48].

#### Acceptability

The acceptability of accelerometer-based assessment of physical activity will be assessed with a modified version of a questionnaire used in previous research [46, 61]. The 11-item acceptability questionnaire used in this study consists of a mix of both Likert-scale quantitative (6) and open-ended qualitative (5) items. The first item assesses relevant previous experience “Have you ever wore a physical activity monitor (e.g., Fitbit) to measure physical activity prior to enrolling in this study?”, ___ no, ___ yes. The next eight items are matched pairs where a quantitative item is paired with a qualitative item to assess a particular aspect of acceptability (i.e., instructions, acceptability of wearing the belt, remembering to wear the belt, integration into daily routine). For example, the second item is “the instructions included in the physical activity monitoring package were easy to follow”, 1 = strongly disagree, 2 = disagree, 3 = neutral, 4 = agree, 5 = strongly agree. Whereas the third item is “If you selected disagree or strongly disagree for the previous item, will you please tell us why the instructions were difficult to follow?”. The penultimate item is “Would you be willing to wear the belt again as a part of a new research study?”, 0 = no, 1 = maybe, 2 = yes. The final item is “What, if anything, would you suggest that we change about how the belt is to be worn in this research study?”

#### Feasibility

The feasibility of accelerometer-based assessment of physical activity within the FFW online intervention will be assessed with descriptive statistics, Pearson’s correlation coefficient, and Bland-Altman analyses. Descriptive statistics will include recruitment rate, eligibility rate, consent rate, participation rates, and retention rates. The mathematical definition for each of these rates will be provided in the “Data analysis” section of this manuscript.

#### Self-reported physical activity

Self-reported physical activity will be measured with the long form of the IPAQ [40, 41]. The long form of the IPAQ is intended for individuals from 15 to 69 years old and purports to measure physical activity in four domains—leisure time, domestic and gardening, work-related, and transport-related—according to the frequency and duration of the physical activity performed in each domain during the previous week. The physical activities measured are separated according to their intensity, which is defined as a distinction between walking, moderate physical activities, and vigorous physical activities. Moderate activities are those that cause a small increase in respiratory frequency and require moderate physical exertion, and vigorous activities cause more breathing than normal, with hard physical exertion [41]. Average minutes per day of MVPA will be calculated based on IPAQ data processing guidelines [62].

#### W1, W2, and W3 survey battery

Instruments designed to measure demographic information, anthropometric characteristics, self-efficacy, and well-being will be included in the survey battery. Data on proposed demographic covariates of well-being [63] and/or physical activity [9] will be collected in the W1 survey battery and will include participant gender, age, race, education-level, marital status, and annual income. Residential zip code data will be collected in the W1 survey battery as a proxy for a host of built environment factors that may be related to an individual’s level of physical activity [9]. Anthropometric data will be assessed in the W1 through the W3 survey battery by asking each participant their height and weight. Demographic, zip code, and anthropometric variables are collectively referred to as covariates from this point forward.

#### Self-efficacy to comply

Participants will be asked to respond to the following item in the W1 survey battery: *How confident are you in your current ability to get yourself to complete at least 30 Fun For Wellness post-introductory challenges within the next four weeks? Thoughtfully completing 30 post-introductory Fun For Wellness challenges may take approximately 4 h.* A five category rating scale structure, where 0 = no confidence, 1 = low confidence, 2 = moderate confidence, 3 = high confidence and 4 = complete confidence, will be implemented for this item, and in all self-efficacy scales from this point forward, based on previous research on effective self-efficacy rating scale structures [64]. Asking participants at the onset of an intervention to make a projection about their compliance with the subsequent intervention is consistent with previous research on compliance [65].

#### Well-being self-efficacy

Well-being self-efficacy has been defined as the extent to which a person believes that they have the ability to achieve a positive state of affairs in important areas of their life [38]. Well-being self-efficacy will be measured at W1 through W3 with the well-being self-efficacy scale (WBSE scale) consistent with both the 2015 FFW efficacy trial [36] and the 2018 FFW effectiveness trial [37]. The 21-item WBSE scale purports to measure seven dimensions of well-being self-efficacy: interpersonal, community, occupational, physical, psychological, economic, and overall. Each of the seven dimensions of well-being self-efficacy purported to be measured by the WBSE scale has a unique item stem that references three different time periods—past (i.e., 30 days ago), present (i.e., right now), and future (i.e., 30 days from now). Evidence for the validity and reliability of scores derived from responses to the original shorter (i.e., 7-item) version of the WBSE scale has been provided [38]. Evidence for the validity and reliability of scores derived from responses to the more recent 21-item version of the WBSE scale, however, is not yet available because the expanded version of the scale is being piloted in the 2018 FFW effectiveness trial.

#### Well-being actions self-efficacy

Well-being actions self-efficacy has been defined as the extent to which a person believes that they have the ability to take actions that may improve the state of affairs in important areas of their life [37]. Well-being actions self-efficacy will be measured at W1 through W3 with the well-being actions self-efficacy scale (WBASE scale) consistent with the 2018 FFW effectiveness trial [37]. The WBASE scale purports to measure six dimensions of well-being actions self-efficacy: interpersonal, community, occupational, physical, psychological, and economic. Each of the six dimensions of well-being actions self-efficacy purported to be measured by the WBASE scale has three items designed to measure it. Evidence for the validity and reliability of scores derived from responses to the WBASE scale is not yet available because the newly developed scale is being piloted in the 2018 FFW effectiveness trial.

#### Physical activity self-efficacy

Physical activity self-efficacy will be measured at W1 through W3 with the physical activity self-efficacy scale (PASE scale) consistent with the 2018 FFW effectiveness trial [37]. The PASE scale is a modified version of the exercise self-efficacy scale [66]. The PASE scale was tailored for the FFW context to assess the extent to which an individual believes that they have the ability to engage in a recommended amount of weekly physical activity for health. The PASE scale is concordant with the IPAQ scale. Specifically, the 48-item PASE scale measures weekly physical activity self-efficacy across four general domains of life: leisure time, domestic and gardening, work-related, and transport-related. Each of the four domains has two unique stems (e.g., how confident are you in your current ability to engage in leisure-related physical activity at a vigorous level of intensity) that reference six increasing time periods (e.g., for at least 10 or 15 or 30 or 45 or 60 or 75 min in the next week). Evidence for the validity and reliability of scores derived from responses to the PASE scale is not yet available because the modified scale is being piloted in the 2018 FFW effectiveness trial.

#### Self-efficacy to regulate physical activity

Self-efficacy to regulate physical activity will be measured at W1 through W3 with the self-efficacy to regulate physical activity scale (SERPA scale) consistent with the 2018 FFW effectiveness trial [37]. The 13-item SERPA scale is a modified version of the barriers self-efficacy scale [67]. The SERPA scale was tailored for the FFW context to assess the extent to which an individual believes that he or she has the ability to overcome possible barriers to engagement in a recommended amount of weekly physical activity for health. Evidence for the validity and reliability of scores derived from responses to the SERPA scale is not yet available because the modified scale is being piloted in the 2018 FFW effectiveness trial.

The reason for including both the SERPA scale and the PASE scale is that the latter scale focuses on an individual’s beliefs in his or her ability to accomplish levels of a task while the former scale focuses on an individual’s beliefs to overcome possible barriers to accomplishing a task that he or she already knows how to do. Self-efficacy theory [8] posits that a self-efficacy level construct (e.g., physical activity self-efficacy) may play a central role in the initiation of a behavior (e.g., engaging in a recommended amount of weekly physical activity) while a self-regulatory efficacy (e.g., self-efficacy to regulate physical activity) may play a central role in the maintenance of a behavior (e.g., engaging in a recommended amount of weekly physical activity over time). The importance of both a self-efficacy level construct and a self-regulatory efficacy construct has been demonstrated in exercise contexts [66, 67].

#### Subjective well-being

Subjective well-being has been defined as an individual’s satisfaction with the state of affairs in important areas of their life [68]. Subjective well-being will be measured at W1 through W3 with the 21-item I COPPE scale [68] consistent with both the 2015 FFW efficacy trial [36] and the 2018 FFW effectiveness trial [37]. The I COPPE scale is concordant with the WBSE scale. Specifically, the seven dimensions of subjective well-being purported to be measured by the I COPPE scale—interpersonal, community, occupational, physical, psychological, economic, and overall—match the seven dimensions of well-being self-efficacy that the WBSE scale was designed to measure. Each of the seven dimensions of subjective well-being purported to be measured by the I COPPE scale is measured with a unique item stem that references three different time periods: past, present, and future. Responses to each item follow an 11-category rating scale structure: from 0 (*worst your life can be*) to 10 (*best your life can be*). Evidence for the validity and reliability of scores derived from responses to the I COPPE scale has been provided [36, 68–70].

#### Well-being actions

The well-being actions construct has been defined as an individual’s actions that may improve the state of affairs in important areas of their life [39]. Well-being actions will be measured at W1 through W3 with the I COPPE actions scale [71] consistent with both the 2015 FFW efficacy trial [36] and the 2018 FFW effectiveness trial [37]. The 18-item I COPPE actions scale is concordant with the WBASE scale. Specifically, the six dimensions of well-being actions purported to be measured by the I COPPE actions scale—interpersonal, community, occupational, physical, psychological, and economic—match the six dimensions of well-being actions self-efficacy that the WBASE scale was designed to measure. Each of the six dimensions of well-being actions purported to be measured by the I COPPE actions scale has three items designed to measure it. Responses to each item follow a 7-category rating scale structure: from 0 (*never*) to 6 (*always*). Evidence for the validity and reliability of scores derived from responses to the original shorter (i.e., 12-item) version of the I COPPE actions scale has been provided [39, 71]. Evidence for the validity and reliability of scores derived from responses to the more recent 18-item version of the I COPPE actions scale, however, is not yet available because the expanded version of the scale is being piloted in the 2018 FFW effectiveness trial.

### Data analysis

Data analyses will include both quantitative and qualitative approaches. Quantitative analyses will be performed in M*plus* 8.0 under maximum-likelihood estimation with robust standard errors [72]. Missing data will be handled with the default approach under the assumption that data are missing at random [73]. When an estimate of a population parameter (e.g., mean difference) is sought, estimation of a 95% confidence interval (CI) will also be sought. When a qualitative approach is taken (e.g., responses to an open-ended acceptability item; feedback from the center staff), feedback will be summarized based on themes that emerge from the research team’s analysis of the feedback. The particular quantitative and/or qualitative methods that will be used to evaluate each specific aim will vary as a function of the focus of the specific aim.

A traffic light system will be used to evaluate results of specific indicators within each specific aim and with regard to the feasibility of a future definitive RCT [43]. Data observed below a lower threshold (i.e., red light) will indicate a potentially serious problem. Data observed above a lower threshold but below an upper threshold (i.e., yellow light) will indicate that caution is warranted. Data observed above an upper threshold (i.e., green light) will indicate support for the feasibility of a future definitive RCT. The particular threshold values that define the traffic light system will vary by the specific indicator within each specific aim (see Table 2).

**Table 2:** Lower and upper bound threshold values that define the traffic light system by aim

Aim	Lower bound threshold	Upper bound threshold
Aim 1		
Acceptability	< 60%	≥ 80%
Recruitment rate	< 40%	≥ 60%
Eligibility rate	< 60%	≥ 80%
Consent rate	< 80%	≥ 90%
Participation rates	< 50%	≥ 70%
Retention rates	< 40%	≥ 60%
Aim 2		
Pearson’s correlation	< .60	≥ .70
Bland-Altman analyses	> 10% of observations beyond M ± 2SD	≤ 5% of observations beyond M ± 2SD
Aim 3		
Acceptability: Quantitative	< 75%	≥ 85%
Acceptability: Qualitative	At least one potentially serious problem	Absence of a potentially serious problem
Aim 4		
Intent to treat	< 0.00	≥ 0.20
Complier average causal effect	< 0.00	≥ 0.20
Indirect effects	Not available	Not available

#### Aim 1

Descriptive statistics will be used to determine if accelerometer-based assessment of physical activity *can* be used within the FFW intervention. Threshold values for the traffic light system for each of the specific indicators that will be used to evaluate aim 1 are based on inferences drawn from the results of previous research [22, 34, 46].

##### Acceptability

Percentage of responses observed in agree or strongly agree to each of the four Likert-scale items designed to assess acceptability will be calculated at wave *W*, where *W* = W1 or W2 or W3. The lower bound threshold is < 60%. The upper bound threshold is ≥ 80%.

##### Recruitment rate

Recruitment rate will be defined as the percentage of patients who select “yes” when asked if they are interested in determining if they are eligible for participation in this study: (*n*_interested_/[*n*_interested_ + *n*_no_ + *n*_defer_]) × 100. The lower bound threshold is < 40%. The upper bound threshold is ≥ 60%.

##### Eligibility rate

Eligibility rate will be defined as the percentage of interested patients who are presented with the informed consent form: (*n*_eligible_/*n*_interested_) × 100. The lower bound threshold is < 60%. The upper bound threshold is ≥ 80%.

##### Consent rate

Consent rate will be defined as the percentage of eligible patients who consent to participate in this study: (*n*_consent_/*n*_eligible_) × 100. The lower bound threshold is < 80%. The upper bound threshold is ≥ 90%.

##### Participation rates

Participation rate at wave *W*, where *W* = W1 or W2 or W3, will be defined as the percentage of consented patients who provide usable data at wave *W*: (*n*_usable data at wave *W*_/*n*_consent_) × 100. The lower bound threshold is < 50%. The upper bound threshold is ≥ 70%.

The definition of usable data will follow the description previously provided in the *ActiGraph wGT3X-BT* section.

##### Retention rates

Retention rate through wave *W*, where *W* = W2 or W3, will be defined as the percentage of consented patients who provide usable data at W1 through wave *W*: (*n*_usable data through wave W_/*n*_consent_) × 100. The lower bound threshold is < 40%. The upper bound threshold is ≥ 60%. The definition of usable data will follow the description previously provided in the *ActiGraph wGT3X-BT* section.

#### Aim 2

Pearson’s correlation coefficient and Bland-Altman analyses will be used to determine if accelerometer-based assessment of physical activity *should* be used within the FFW intervention. Threshold values for the traffic light system for the specific indicators that will be used to evaluate aim 2 are based on inferences drawn from the results of previous research [47, 48].

##### Pearson’s correlation

The linear relationship between the estimates of average minutes per day of MVPA at wave *W*, where *W* = W1 or W2 or W3, produced by the ActiGraph wGT3X-BT, the Fitbit Zip, and the IPAQ, will be estimated with Pearson’s correlation [74]. The lower bound threshold is < .60. The upper bound threshold is ≥ .70.

##### Bland-Altman analyses

Agreement between the estimates of average minutes per day of MVPA at wave *W*, where *W* = W1 or W2 or W3, produced by the ActiGraph wGT3X-BT, the Fitbit Zip, and the IPAQ, will be evaluated with Bland-Altman analyses [75]. First, to test for a systematic difference between pairs of estimates and zero at wave *W*, a paired *t* test will be conducted. Second, to test for proportional bias at wave *W*, a linear regression will be conducted where the difference between pairs of estimates will be regressed on the mean of the two estimates. Third, the Bland-Altman plot will be constructed at wave *W*, where the difference between pairs of estimates is represented on the *y*-axis, the mean of the two estimates is represented on the *x*-axis, and three horizontal lines are depicted with regard to the *y*-axis: *M* + 2SD, *M*, *M* − 2SD. The traffic light system will be based on the Bland-Altman plot and not on a result from a hypothesis test [42–44]. The lower bound threshold is > 10% of observations beyond *M ±* 2SD. The upper bound threshold is ≤ 5% of observations beyond *M ±* 2SD.

#### Aim 3

A descriptive statistic and qualitative data will be used to determine *how* to implement accelerometer-based assessment of physical activity within the FFW intervention.

Threshold values for the traffic light system for the specific indicators that will be used to evaluate aim 3 are based on inferences drawn from the results of previous research [46].

##### Acceptability: quantitative

Percentage of responses observed in “yes” to the following item—“Would you be willing to wear the belt again as a part of a new research study?” will be calculated at wave *W*, where *W* = W1 or W2 or W3. The lower bound threshold is < 75%. The upper bound threshold is ≥ 85%.

##### Acceptability: qualitative

Themes that emerge from responses to the five qualitative items designed to assess acceptability will be analyzed at wave *W*, where *W* = W1 or W2 or W3. Possible improvements will be considered with regard to the protocol for accelerometer-based assessment of free-living physical activity within the FFW online intervention among adults with obesity in the USA. Specifically, improvements will be considered with regard to instructions for wearing the belt, acceptability of wearing of the belt, remembering to wear the belt, integration of wearing the belt into the daily routine, and why some participants may be unwilling to wear the belt as a part of new research study. The lower bound threshold is the presence of at least one potentially serious problem with the accelerometer-based protocol that is unable to be addressed in a future study. The upper bound threshold is the absence of a potentially serious problem with the accelerometer-based protocol that is unable to be addressed in a future study.

#### Aim 4

Inferential statistical models under both an intent to treat (ITT) approach and a complier average causal effect (CACE) approach will be used to provide a preliminary effect size estimate for each direct effect depicted in the conceptual model (see Fig. 2) for the FFW online intervention (e.g., FFW → Physical Activity) at wave W, where W = W2 or W3. Using both an ITT approach and a CACE approach is consistent with data analyses from the 2015 FFW efficacy trial [36, 38, 39] and with the data analysis plan for the 2018 FFW effectiveness trial [37]. Covariates, the outcome at W1, and group assignment will be specified as predictors in both approaches. Threshold values for the traffic light system for the specific indicators that will be used to evaluate aim 4 are based on commonly used heuristics for Cohen’s *d* [76].

##### ITT

The ITT approach will estimate each direct effect of being assigned to the FFW intervention at wave *W*, where *W* can equal W2 or W3 [77]. The lower bound threshold is < 0.00. The upper bound threshold is ≥ 0.20.

##### CACE

The CACE approach will estimate each direct effect of complying with the FFW intervention [78–81]. The lower bound threshold is < 0.00. The upper bound threshold is ≥ 0.20.

##### Indirect effects

At this time, we do not plan to provide a preliminary effect size estimate for the indirect effects of FFW online intervention depicted in the conceptual model (see Fig. 2) for the FFW online intervention (e.g., FFW → Physical Activity Self-Efficacy → Physical Activity) due to a methodological limitation. Specifically, we are unaware of any published methodological work on the estimation of an indirect effect and its standard error within the CACE framework. For this reason, the general data analytic approach taken (or planned to be taken) in both the 2015 FFW efficacy trial [36, 38, 39] and the 2018 FFW effectiveness trial has been to first evaluate evidence for each direct (or equivalently, overall) effect before possibly investigating evidence for the possible decomposition of an overall effect into indirect and direct effects [82] in the future should relevant methodological advancements become available.

## Discussion

FFW is an online behavioral intervention designed to promote growth in well-being and physical activity by providing capability-enhancing learning opportunities to participants. The objective of this manuscript is to describe the protocol for a feasibility study designed to address uncertainties regarding the inclusion of accelerometer-based assessment of free-living physical activity within the FFW intervention among adults with obesity in the USA. The protocol described in this paper logically builds upon both the 2015 FFW efficacy trial [36] and the 2018 FFW effectiveness trial [37]. Results from the feasibility study described in this paper are intended to inform the preparation of a future definitive RCT. Like the protocol for every feasibility study, however, the protocol for the feasibility study described in this paper has both strengths and weaknesses.

We are aware of at least four notable strengths for the feasibility study described in this paper. First, the study described in this paper seeks to address a major limitation found in both the 2015 FFW efficacy trial and the 2018 FFW effectiveness trial: measurement of physical activity via self-report only. We believe that providing some initial evidence for the feasibility of including accelerometer-based assessment of physical activity within FFW may be an important next step in the continual development of the intervention, particularly given recent findings that suggest less than high agreement between estimates of physical activity based on self-report versus accelerometer-based [30]. Second, the feasibility study described in this paper attempts to build an evidence-based foundation for a specific response to a global need for readily scalable online behavioral interventions that effectively promote physical activity in adults [16]. We believe that the FFW online behavioral intervention may have the potential to eventually become useful, in some small but important way given the magnitude of the problem, in responding to the global pandemic of physical inactivity [13, 14]. Third, the feasibility study described in this paper attempts to build an evidence-based foundation for a specific response to the troubling global trend toward obesity [1]. We believe that the FFW intervention, because of its conceptual basis in self-efficacy theory, may be effective in promoting physical activity in obese adults [6, 32, 33]. Finally, the feasibility study described in this paper aligns well with recent recommendations put forth by the Community Preventive Services Task Force [33]. More specifically, the feasibility study described in this paper proposes a physical activity intervention for adults with obesity (i.e., FFW) that includes activity monitors (i.e., accelerometers) and promotes physical activity within a more broadly focused weight management program where there is access to a health care provider (i.e., at a local bariatric service center within a major healthcare organization in the Midwest of the USA).

We are aware of at least three notable limitations for the feasibility study described in this paper. The first limitation is that recruitment of participants is to occur within a relatively controlled local context. While recruiting participants at a local bariatric service center within a major healthcare organization in the Midwest of the USA will afford the research staff the opportunity to follow up with both center staff and participants during the course of the study to address unforeseen areas of uncertainty, it also may limit the generalizability of the results of the study [42–44]. Future research that evaluates the feasibility of accelerometer-based assessment of physical activity within the FFW online behavioral intervention in a less controlled context (e.g., an online survey panel company) may be worthwhile given the scientific utility of evaluating interventions in a variety of contexts [83].

The second limitation is that each participant in this study will be determined to be eligible for this study based on values provided by self-report. For some eligibility criteria this limitation may be regarded as relatively minor due to a structural characteristic of the study design. For instance, the eligibility criterion that BMI ≥ 25.00 kg/m^2^ should be truly met (at least at baseline) because enrollment in the preoperative weight management program provided by the center (from which participants in this study will be recruited) requires BMI ≥ 35.00 kg/m^2^ or ≥ 40.00 kg/m^2^ depending on other health indicators. Similarly, the eligibility criterion that a participant has the ability to access the online intervention may be viewed as reasonably likely to be truly met in at least most cases for a couple of reasons. First, the Pew Research Center estimates that more than three quarters of the adult population in the USA currently own a smartphone [84], a device that should be able to access the online activities that will occur in this study. Second, the login credential verification that occurs at the end of the recruitment phase of this study requires a participant to respond to a message sent to the e-mail account provided (i.e., establishing an online communication pathway) before proceeding to the eligibility determination phase of this study. Finally, the eligibility criterion that a participant communicates an honest willingness to comply with instructions for physical activity monitoring may be viewed as likely to be met in at least most cases for a couple of reasons. First, the compliance rates for observed in studies with similar physical activity monitoring protocols, such as the 2003–2004 NHANES study [22] and the REGARDS study [34], generally were quite high. Second, the accelerometer devices will provide objective indicators (e.g., wear time) of the observed compliance with the instructions for physical activity monitoring. For two eligibility criteria (i.e., age and the absence of simultaneous enrollment in another intervention program promoting either well-being or physical activity), however, this self-report-based limitation should be regarded as potentially more problematic due to the absence of a structural characteristic in the study design that guards against the provision of false information. Most generally, a limitation of this study is that it is possible one or more participants in this study may provide false information that leads to an incorrect decision regarding their true eligibility for enrollment in this study.

The third limitation deals with some uncertainty regarding the qualitative approach taken in aim 3. While the qualitative approach taken in aim 3 to assess the acceptability of accelerometry within the FFW intervention (i.e., open-ended questions) represents an extension of a more quantitatively focused questionnaire used in previous research [46, 61], it may still fail to capture at least some important information that a more rigorous qualitative approach (e.g., in-depth interviews) may provide. If the qualitative approach taken in aim 3 of this study fails to provide adequate information to reasonably assess the acceptability of accelerometry within the FFW intervention, then subsequent research may need to dedicate more resources (e.g., staffing to conduct in-depth interviews) to support a more rigorous qualitative approach to more fully investigate aim 3.

## Additional file

Additional file 1 SPIRIT 2013 Checklist: Recommended items to address in a clinical trial protocol and related documents. (DOC 122 kb)

## Abbreviations

BET I CAN: Behaviors, emotions, thoughts, interaction, context, awareness next steps
BMI: Body mass index
CA: California
CACE: Complier average causal effect
FFW: Fun for Wellness
FL: Florida
I COPPE: Interpersonal, community, occupational, physical, psychological, economic
IPAQ: International physical activity questionnaire
ITT: Intent to treat
*M*: Mean
MVPA: Moderate to vigorous physical activity
NHANES: National Health and Nutritional Examination survey
PAMP: Physical activity monitoring package
PASE: Physical activity self-efficacy
RCT: Randomized controlled trial
REGARDS: Reasons for Geographic and Racial Differences in Stroke
*SD*: Standard deviation
SERPA: Self-efficacy to regulate physical activity
UC: Usual care
USA: United States of America
W1: Wave 1
W2: Wave 2
W3: Wave 3
WBASE: Well-being actions self-efficacy
WBSE: Well-being self-efficacy
WHO: World Health Organization
